# Cardiac Manifestations in Fabry Disease: A Case Report on Two Siblings

**DOI:** 10.3390/diagnostics15030340

**Published:** 2025-01-31

**Authors:** Slavica Kovačić, Tin Nadarević, Petar Žauhar, Božidar Vujičić, Iva Žuža

**Affiliations:** 1 Department of Diagnostic and Interventional Radiology, University Hospital Centre Rijeka, 51000 Rijeka, Croatia; tin.nadarevic@gmail.com (T.N.); petar.zauhar@gmail.com (P.Ž.); iva.zuza276@gmail.com (I.Ž.); 2 Faculty of Medicine, University of Rijeka, 51000 Rijeka, Croatia; vujicic.bozidar@gmail.com; 3 Department of Nephrology, Dialysis and Transplantation, University Hospital Centre Rijeka, 51000 Rijeka, Croatia

**Keywords:** Anderson-Fabry disease, cardiac magnetic resonance imaging, T1-mapping

## Abstract

**Background/objectives:** Anderson-Fabry disease (FD) is a rare hereditary disorder caused by deficient alpha-galactosidase A activity, which leads to multisystemic complications, including significant cardiac involvement. In this case report, we describe two siblings with distinct cardiac manifestations of FD. **Methods**: The medical data of two siblings who were managed and treated at a tertiary hospital center in Croatia were obtained by detailed analysis of electronic medical records. All available data were structured in chronological order. **Results**: A 42-year-old male with chronic renal failure and severe left ventricular hypertrophy (LVH) was diagnosed with FD during testing for inclusion on the kidney transplant waiting list. The diagnosis was confirmed by cardiac magnetic resonance imaging (CMR), which revealed non-ischemic fibrosis typical of FD. Following enzyme replacement therapy (ERT), he underwent a successful kidney transplantation. The second case describes the 36-year-old brother, who was diagnosed through family screening and, despite normal initial cardiac ultrasound findings, exhibited early cardiac involvement through reduced T1-mapping values. Immediate initiation of ERT led to normalization of T1 values and successful renal transplantation. **Conclusions**: This report underscores the importance of family screening and early diagnosis in FD and highlights the role of CMR in detecting preclinical cardiac involvement.

## 1. Introduction

Anderson-Fabry disease (FD) is a rare, multisystemic X-linked lysosomal storage disorder caused by altered activity of the lysosomal enzyme alpha-galactosidase A (αGalA), which leads to the accumulation of pathological substrate in lysosomes. The resulting cellular dysfunction triggers a cascade that includes inflammation, oxidative stress, cellular death, small vessel injury, tissue ischemia, and the development of cardiac and renal fibrosis [1,2]. FD can be reliably diagnosed in most males by deficient or absent αGALA activity in the plasma or peripheral leukocytes, whereas in most females, molecular testing should be performed to detect the respective gene mutation, as heterozygous patients might have regular αGALA enzyme activity [3]. The multisystemic and non-specific nature of FD symptoms and medical professionals’ lack of awareness can lead to significant delays in diagnosis. Screening of high-risk populations, such as patients with chronic kidney disease (CKD) or suspected newborns, along with their family members, facilitates the timely identification of patients in the early stages of FD and thus enables the immediate initiation of treatment [4].

The incidence of FD is challenging to determine due to underdiagnosis in clinical practice and/or overestimation in screening programs and is reported to be 1:40,000–117,000 [2,3,4,5]. Most patients with FD present with renal insufficiency with progressive glomerulosclerosis and proteinuria that does not respond to enzyme replacement therapy [6]. The most common initial symptoms of FD are neurological—neuropathic pain is the most common clinical complaint in patients with FD. These initial symptoms may already occur during childhood.

Although FD is a multi-systemic disease, it can be primarily considered a cardiac condition in terms of mortality, with most fatalities classified as ‘sudden cardiac death’ [7]. Progressive left ventricular hypertrophy (LVH) is the most common cardiac manifestation, reported to be up to 88% in males with FD over the age of 30 and in more than a third of women older than 40 years [8]. Further cardiac abnormalities include chronotropic incompetence, sinus node dysfunction and severe atrioventricular block, resting bradycardia, and impaired heart rate response on exertion [2].

Subclinical cardiac involvement may be the first sign of organ damage in patients with FD. Cardiac magnetic resonance imaging (CMR) is essential for early diagnosis and staging of cardiac FD. It enables accurate, non-invasive assessment of LVH and myocardial fibrosis as well as glycosphingolipid deposition in the myocardium, which occurs before the development of significant LVH [9,10]. Recent studies have shown that CMR T1-mapping sequences allow the characterization of the myocardium by identifying myocardial edema, lipid accumulation, and extracellular volume expansion in myocytes, which can detect early cardiac involvement. Some limitations have emerged in clinical practice, such as the progressive pseudo-normalization of T1 relaxation times occurring in later stages, characterized by inflammation, hypertrophy, and fibrosis, as well as the need for the standardization of values according to the imaging equipment and protocols in use [11,12,13].

This article presents two siblings with different cardiac manifestations of FD.

## 2. Case Presentation

**Figure 1:**
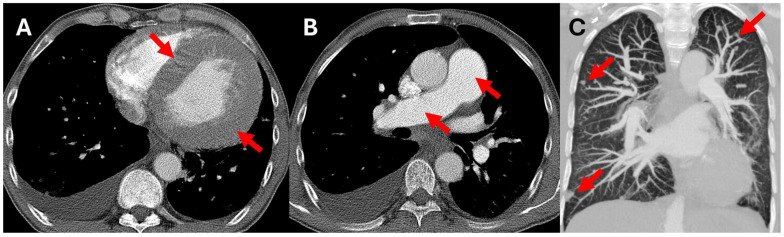
Contrast-enhanced CT pulmonary angiography in mediastinal (**A**,**B**) and lung (**C**) window. (**A**)—left ventricular hypertrophy (arrows), (**B**)—dilated main and right pulmonary artery (arrows), (**C**)—intrapulmonary nodules (arrows).

We present a 42-year-old male patient who was referred to our institution (University Hospital Center Rijeka, Rijeka, Croatia) for a nephrology consult regarding a kidney transplant. The patient had already been diagnosed with chronic renal failure and had been on hemodialysis for 7 years. He was first diagnosed with chronic renal failure 19 years ago with an explanation of a severe case of glomerulonephritis. During one session of hemodialysis at our facility, the patient reported acute onset of severe dyspnea, which prompted the order of CT pulmonary angiography with clinical suspicion of pulmonary thromboembolism (PTE). No evidence of PTE was found. However, other findings reported included left ventricular myocardial hypertrophy, dilated pulmonary arteries, and several intrapulmonary nodules suspicious for intrapulmonary metastases (Figure 1). Further diagnostic workup included a thorax, abdomen, and pelvis CT scan, which showed no suspicion of a primary malignancy.

**Figure 2:**
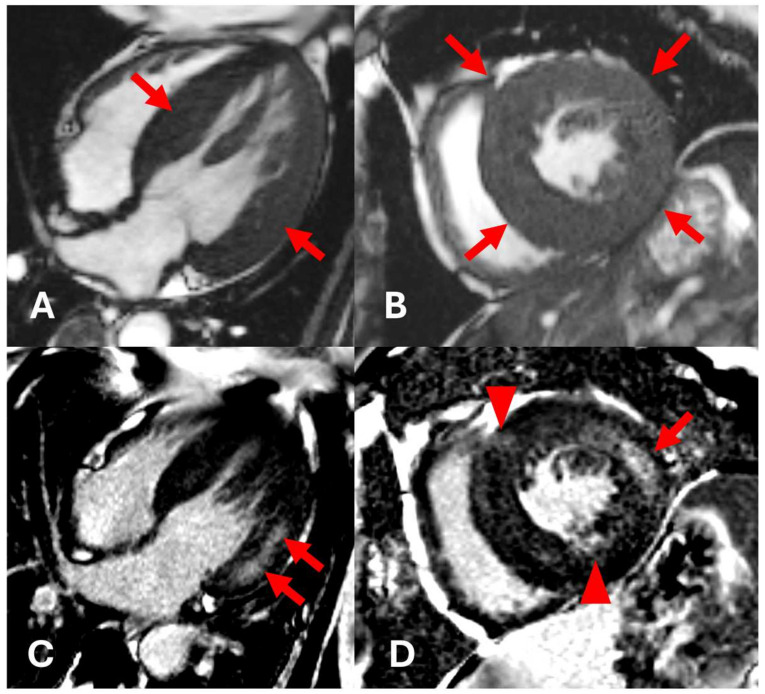
Cardiac magnetic resonance (CMR) imaging; native cine 4-chamber (4ch) view and short-axis (SA) view (**A**,**B**). Late gadolinium enhancement (LGE) 4ch and SA views (**C**,**D**). (**A**,**B**)—Concentric left ventricular myocardium hypertrophy (arrows). (**C**)—LGE 4ch view shows patchy fibrosis in the mid-myocardial part of the lateral wall (arrows), typical for Fabry disease. (**D**)—LGE SA view shows patchy fibrosis in the lateral wall (arrow) and patchy fibrosis in anteroseptal and inferoseptal segments (triangles).

**Figure 3:**
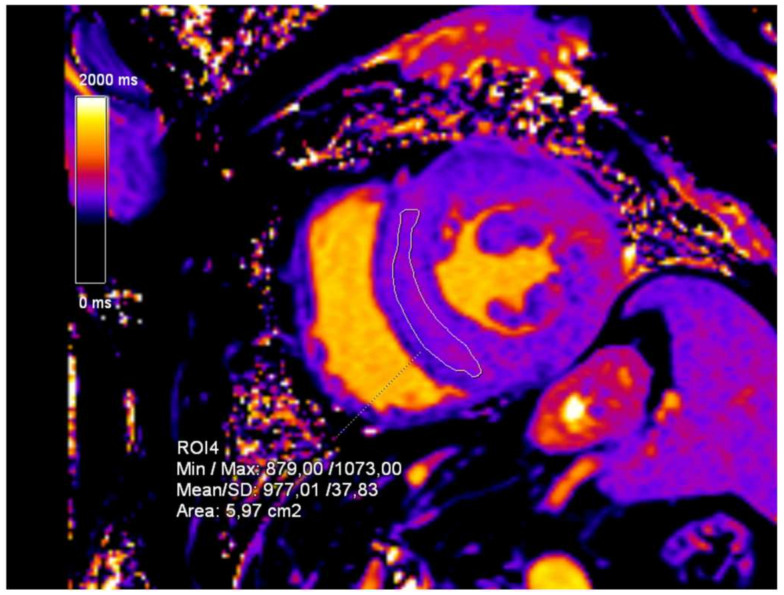
T1-mapping sequence, short-axis (SA) view. The mean T1-mapping value of the left ventricle myocardium is 977 ms.

An initial cardiac ultrasound was performed, which confirmed the presence of myocardial hypertrophy. The report stated concentric myocardial hypertrophy with impaired systolic function (left ventricular ejection fraction of 43%) and signs of pulmonary hypertension. An initial CMR exam was ordered and showed concentric myocardial hypertrophy of the left ventricle with non-ischemic fibrosis in the basal and mid segments of the lateral wall and patchy non-ischemic fibrosis in the mid-anteroseptal and inferoseptal segments (Figure 2). CMR T1-mapping was also performed, and the values measured 977 ms, whilst the normal reference values were considered to be 950 ± 20 ms (Figure 3).

The results of CMR were highly suggestive of FD. The patient was tested for αGALA enzyme deficiency and changes in the GLA gene during further diagnostic work-up, confirming the FD diagnosis. A classical type mutation c.[540G>C] (p.[L 180F]) was found. Further testing revealed a low alpha-galactosidase activity of 0.02 nmol/spot/21h (reference interval >0.185 nmol/spot/21h) and elevated lyso-Gb3 12.1 ng/mL (reference interval ≤ 1.8 ng/mL) [14]. These findings facilitated further family screening.

**Figure 4:**
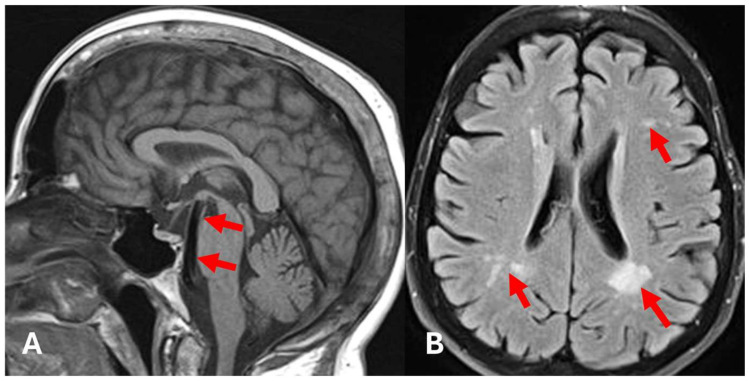
Brain MRI, T1 sequence, sagittal view (**A**), FLAIR sequence, axial view (**B**). (**A**)—Thick tubular flow-void located ventral to the pons and mesencephalon (arrows)—dolichoectatic basilar artery. (**B**)—White matter hyperintensities due to small vessel disease (arrows).

An MRI of the brain was also performed to detect any signs of brain manifestations of FD. The exam showed basilar artery ectasia and mild to moderate deep white matter changes due to small vessel disease related to FD (Figure 4).

Following the diagnosis and completing the diagnostic work-up, the patient was immediately started on agalsidase beta—a recombinant human αGALA enzyme replacement therapy (ERT). Four years after the first hospitalization at our institution, the patient underwent a kidney transplant with satisfactory results.

**Figure 5:**
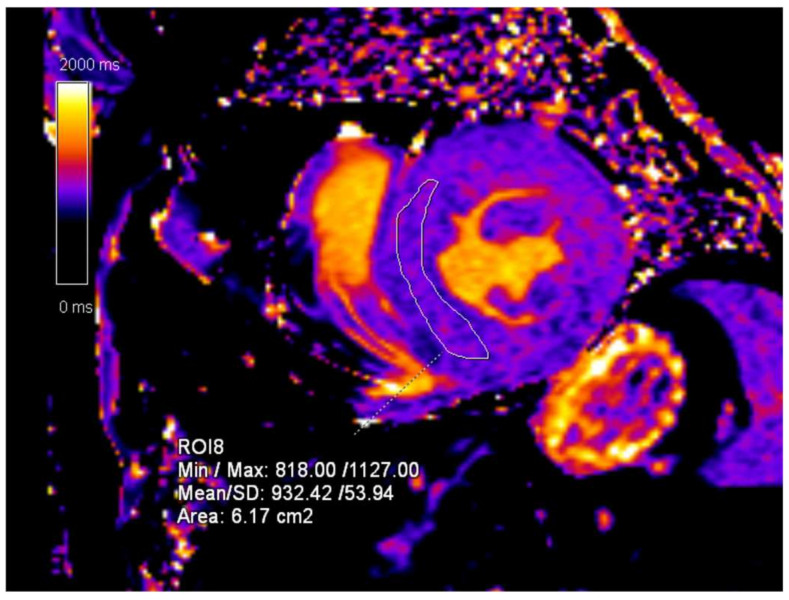
CMR follow-up 24 months after the initial exam. T1-mapping sequence, short-axis (SA) view. The mean T1-mapping value of the left ventricle myocardium is 932 ms.

The follow-up CMR was performed 12 months after the initial exam, and the progression of LVH was reported with no signs of obstruction or right ventricular hypertrophy (RVH). An increase in T1-mapping values, now measuring 1032 ms, was noted. Eighteen months after the initial CMR, a normalization of the T1-mapping values was observed, which were reported to be 950 ms, a value within the reference range. The final CMR exam was performed 24 months after the initial exam showed no signs of disease progression, with T1-mapping values within the normal range (932 ms) (Figure 5).

**Figure 6:**
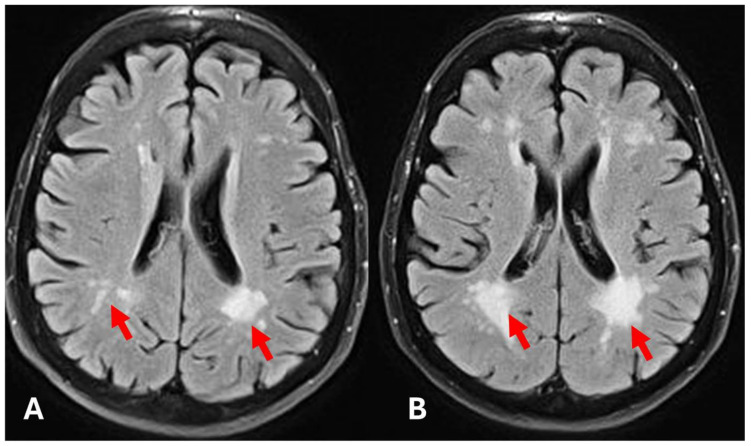
Initial brain MRI (**A**), follow-up brain MRI 78 months after the initial exam (**B**). Significant progression of white matter hyperintensities due to the progression of small vessel disease (arrows).

Subsequent MRI scans of the brain showed a gradual progression of bilateral white matter changes. The first progression was detected 60 months after the initial brain MRI, and 78 months after the initial exam, the exam showed severe white matter changes due to the progression of small vessel disease related to FD (Figure 6).

**Figure 7:**
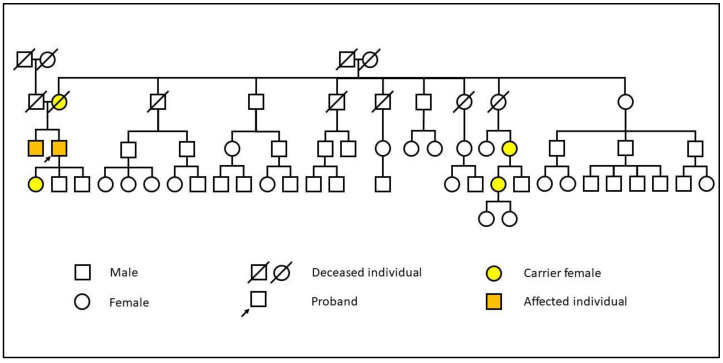
Family pedigree of the presented siblings. The proband is the first case (older brother).

A systematic family screening was initiated after the FD diagnosis in the previously presented case. The patient’s mother was determined to be the gene carrier, but she exhibited no manifestations of FD. The mother had five brothers and three sisters, of whom three brothers and two sisters are deceased, and all have children of their own (Figure 7).

Here, we present the patient’s brother—a 36-year-old male who was previously diagnosed with chronic kidney disease (CKD) with secondary hyperparathyroidism and arterial hypertension. The patient was also diagnosed with the same gene mutation—classic type mutation c.[540G>C] (p.[L 180F]). This patient also exhibited low alpha-galactosidase activity of 0.03 nmol/spot/21h (reference interval > 0.185 nmol/spot/21h) and elevated lyso-Gb3 6.0 ng/mL (reference interval ≤ 1.8 ng/mL).

**Figure 8:**
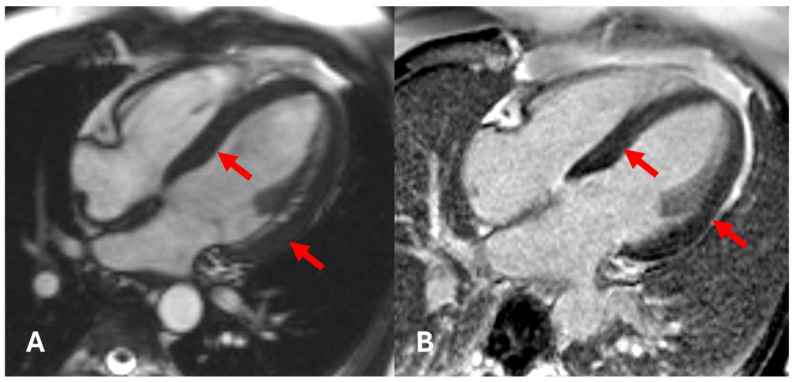
Initial CMR. Cine sequence in 4ch view (**A**) and LGE sequence in 4ch view (**B**). Normal thickness of the left ventricular myocardium, with no fibrosis on LGE sequences (arrows).

**Figure 9:**
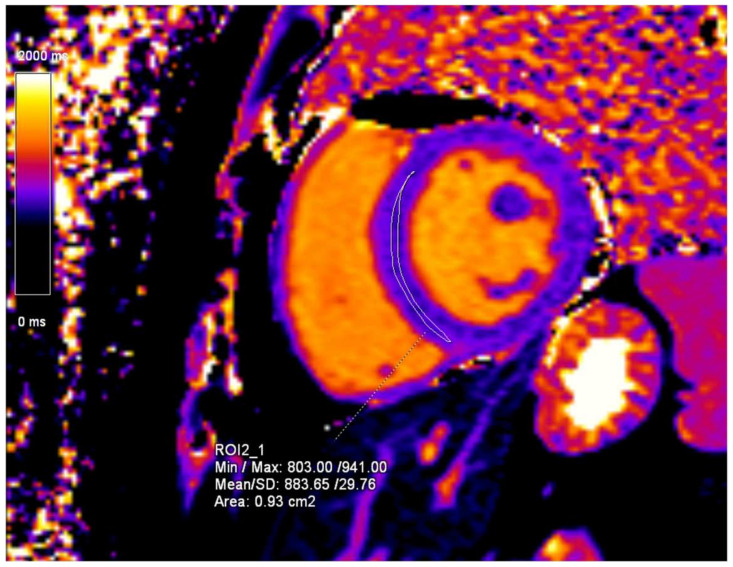
T1-mapping sequence, short-axis (SA) view. The mean T1-mapping value of the left ventricle myocardium is 884 ms.

Initially, a cardiac ultrasound was performed, which, unlike that of his brother, showed normal findings. The initial CMR examination showed normal ejection fraction and myocardial contractility with no myocardial hypertrophy (Figure 8). The only findings indicative of cardiac manifestation of FD were CMR T1-mapping values, which were measured to be 884 ms (Figure 9).

**Figure 10:**
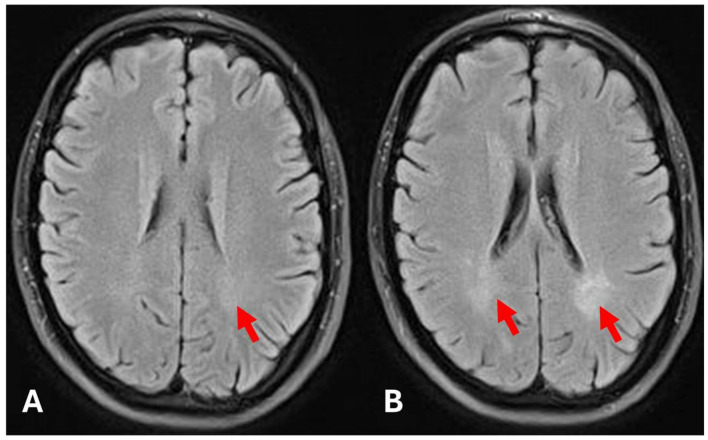
Initial brain MRI (**A**), follow-up brain MRI 78 months after the initial exam (**B**). (**A**)—Very mild white matter hyperintensities due to small vessel disease in the left cerebral hemisphere (arrow). (**B**)—Significant progression of white matter hyperintensities (arrows).

Brain MRI was performed as part of a routine examination and showed very mild white matter changes adjacent to lateral ventricles. Other findings were unremarkable, and other signs of Fabry disease were not present (Figure 10).

The patient was promptly started on agalsidase beta and placed on the waiting list for a kidney transplant. A successful kidney transplant was performed nine months after the initial diagnosis.

**Figure 11:**
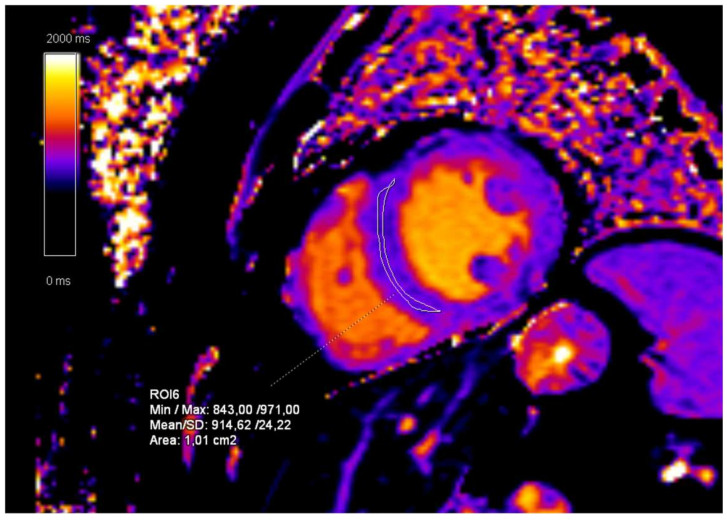
CMR follow-up 24 months after the initial exam. T1-mapping sequence, short-axis (SA) view. The mean T1-mapping value of left ventricle myocardium is 915 ms.

The follow-up CMR performed 24 months after the initial diagnosis showed a slight volumetric increase of the left ventricle with preserved ejection fraction and a significant increase in the T1-mapping values (938 ms) (Figure 11). The further CMR performed 48 months after the initial diagnosis showed no signs of progression, now with left ventricular volumetry values in the reference range and T1-mapping values in the normal reference range (915 ms). The last MRI exam was performed 60 months after the initial exam and showed moderate white matter changes (Figure 10).

## 3. Discussion

The epidemiological variability of FD may be influenced by underdiagnosis and misdiagnosis due to the variable clinical presentation and the lack of awareness of the disease among healthcare professionals. The heterogeneous clinical presentation, especially in the early stages, further complicates diagnosis, which delays the initiation of treatment that can significantly alter the course of the disease and improve quality of life [15]. As shown in our case report, family screening plays a crucial role in the early diagnosis of FD. Studies have shown how cascade family screening can lead to an average of five or more newly diagnosed patients who are more likely to be diagnosed at early stages of the disease [16]. Our second case demonstrated the practical application of this principle; however, fully implementing family screening took a lot of work due to the various clinical centers involved in treating and managing all family members.

The necessity of cardiological workup for cardiovascular disease in patients with CKD is well known due to the complex interplay of the two entities. Still, the situation is further complicated in patients with FD. A study conducted at Mount Sinai School of Medicine re-evaluated multiple publications on the prevalence of FD in the screening of “high-risk” populations and showed that the prevalence of FD in patients undergoing hemodialysis may be 0.21% in men and 0.15% in women, while studies conducted in patients undergoing renal transplantation found pathogenic mutations associated with FD in 0.24% of male patients but in none of the female patients. This association was critical to the patient’s diagnosis in the first case, further emphasizing the importance of screening in “high-risk” populations. In addition, the same study highlighted the importance of screening for FD in patients with otherwise unexplained LVH or HCM, as this symptom was found in up to 0.94% of male and 0.90% of female patients with pathogenic FD mutations [17].

Research into the use of CMR for screening FD has gained importance due to its effectiveness in detecting early cardiac involvement, which is crucial for timely therapeutic intervention and significantly affects treatment outcomes [18,19,20]. One advantage of CMR is its superior accuracy in quantifying left ventricular mass (LVM) and assessing myocardial architecture. Studies have shown that transthoracic echocardiography often overestimates LVM and has lower reproducibility than CMR, affecting the diagnostic and therapeutic approach [18,21]. In addition, CMR LGE can discern atypical patterns of cardiac involvement, particularly in the inferolateral wall of the left ventricle, always sparing at least a thin portion of the subendocardial and subepicardial layer, which distinguishes FD from other infiltrative cardiomyopathies such as amyloidosis and sarcoidosis [22]. Our findings in the first case with the delayed diagnosis of FD meet these criteria, whereas in the second case, no such findings were shown, mainly due to the early stage of the disease. The differences in myocardial fibrosis patterns between siblings can be attributed to the age difference (over five years), which reflects different degrees of progression of Fabry disease. Secondly, the older brother (first case) had already been treated with hemodialysis for seven years, which also affects the remodeling of the myocardium due to volume load. Similar disparities in renal and neurological damage were probably caused by the late start of treatment of the disease with enzyme replacement therapy [23]. The importance of LGE in FD is also reflected in its role as a significant prognostic marker and in monitoring treatment response, particularly ERT [24,25]. In addition to LGE, CMR techniques such as T1 mapping can further improve Fabry disease’s diagnostic and predictive capabilities. T1-mapping quantifies the T1 relaxation time of myocardial tissue. It detects changes in myocardial composition that precede visible morphological changes, making it an essential tool for early diagnosis and monitoring disease progression. The typical finding in patients with FD is low T1-mapping values due to an accumulation of sphingolipids in the myocardial tissue, a pathological finding that precedes the development of LVH [26]. Another possibility provided by T1-mapping in FD is to follow the patient’s response to ERT. Studies show that successful ERT can increase T1-mapping levels, reflecting a reduction in substrate accumulation, so T1-mapping can be used as a biomarker for evaluating ERT’s efficacy without using a contrast medium [1]. However, normalization of T1-mapping values may also reflect disease progression rather than solely the effects of ERT, a phenomenon known as “pseudo-normalization,” where T1 values appear to normalize despite ongoing disease progression due to underlying fibrosis and lipid accumulation [27]. In addition, the presence of myocardial trabeculations can also lead to higher T1-mapping values. Trabeculations are associated with early glycosphingolipid deposition and may serve as an early marker of FD prior to more severe myocardial fibrosis and dysfunction. All things considered, the interpretation of changes in T1-mapping values involves the complex relationship between myocardial fibrosis, glycosphingolipid accumulation, and the potential for “pseudo-normalization” such that continued monitoring and comprehensive assessment remain essential for accurate evaluation of cardiac involvement and disease progression in all patients with FD [28]. Results consistent with these were shown primarily in the second case, in which the initially low T1-mapping values normalized after the initiation of ERT. The difference in T1 values between the two cases presented was most likely due to the early detection of FD in the second patient, which could have resulted in a better response to ERT.

Although CMR possesses advantages in detecting early disease, its incorporation in screening protocols may not be feasible due to the complexity of the exam, availability, and potential lack of cost-effectiveness. However, the capability of CMR to deliver detailed insights into myocardial structure and function establishes it as an invaluable tool in evaluating the diagnosis, risk prognosis, and therapy response tracking in FD.

## 4. Conclusions

In this case report, we demonstrated the significant impact of CMR on initial diagnosis and treatment decisions in two brothers with FD at different stages of the disease. Apart from the benefit of the initial diagnostic work-up, we also highlight the role of CMR in the follow-up of patients with FD, as well as monitoring myocardial damage, disease progression, and therapeutic effects of ERT. The first case represented an advanced form of FD, showing marked myocardial hypertrophy. CMR played an essential role in assessing the extent of cardiac involvement, with LGE as a prognostic marker for disease severity. The second case exhibited a markedly different clinical presentation. Initial transthoracic echocardiography showed no signs of LVH. However, with reduced T1-mapping values, we demonstrated initial, preclinical cardiac involvement due to an accumulation of sphingolipids in the myocardium. Furthermore, we could follow the normalization of T1-mapping values once the patient started ERT.

Finally, we also highlight the importance of family screening in diseases such as FD and the screening of high-risk populations, which enables early diagnosis and leads to better clinical outcomes and quality of life for patients.

## Data Availability

The original contributions presented in the study are included in the article, further inquiries can be directed to the corresponding author.

## References

[1] Rajan J.N., Ireland K., Johnson R., Stepien K.M. (2021). Review of Mechanisms, Pharmacological Management, Psychosocial Implications, and Holistic Treatment of Pain in Fabry Disease. J. Clin. Med..

[2] Hagège A., Réant P., Habib G., Damy T., Barone-Rochette G., Soulat G., Donal E., Germain D.P. (2019). Fabry disease in cardiology practice: Literature review and expert point of view. Arch. Cardiovasc. Dis..

[3] Germain D.P. (2010). Fabry disease. Orphanet J. Rare Dis..

[4] Battaglia Y., Fiorini F., Azzini C., Esposito P., De Vito A., Granata A., Storari A., Mignani R. (2021). Deficiency in the Screening Process of Fabry Disease: Analysis of Chronic Kidney Patients Not on Dialysis. Front. Med..

[5] Spada M., Pagliardini S., Yasuda M., Tukel T., Thiagarajan G., Sakuraba H., Ponzone A., Desnick R.J. (2006). High Incidence of Later-Onset Fabry Disease Revealed by Newborn Screening. Am. J. Hum. Genet..

[6] Faro D.C., Di Pino F.L., Monte I.P. (2024). Inflammation, Oxidative Stress, and Endothelial Dysfunction in the Pathogenesis of Vascular Damage: Unraveling Novel Cardiovascular Risk Factors in Fabry Disease. Int. J. Mol. Sci..

[7] Baig S., Edward N.C., Kotecha D., Liu B., Nordin S., Kozor R., Moon J.C., Geberhiwot T., Steeds R.P. (2018). Ventricular arrhythmia and sudden cardiac death in Fabry disease: A systematic review of risk factors in clinical practice. EP Eur..

[8] Linhart A., Kampmann C., Zamorano J.L., Sunder-Plassmann G., Beck M., Mehta A., Elliott P.M., on Behalf of European FOS Investigators (2007). Cardiac manifestations of Anderson-Fabry disease: Results from the international Fabry outcome survey. Eur. Heart J..

[9] Hiestand R., Nowak A., Sokolska J.M., Chan R., Ruschitzka F., Manka R., Gruner C. (2023). Clinical and CMR characteristics associated with cardiac events in patients with Fabry disease. Int. J. Cardiol..

[10] Pieroni M., Moon J.C., Arbustini E., Barriales-Villa R., Camporeale A., Vujkovac A.C., Elliott P.M., Hagege A., Kuusisto J., Linhart A. (2021). Cardiac Involvement in Fabry Disease: JACC Review Topic of the Week. J. Am. Coll. Cardiol..

[11] Ricci F., Bisaccia G., Mansour D., Molinari L.V., Di Mauro M., Renda G., Khanji M.Y., Gallina S. (2023). Prognostic Significance of Late Gadolinium Enhancement in Fabry Disease—A Systematic Review and Meta-Analysis. Am. J. Cardiol..

[12] Messroghli D.R., Moon J.C., Ferreira V.M., Grosse-Wortmann L., He T., Kellman P., Mascherbauer J., Nezafat R., Salerno M., Schelbert E.B. (2016). Clinical recommendations for cardiovascular magnetic resonance mapping of T1, T2, T2* and extracellular volume: A consensus statement by the Society for Cardiovascular Magnetic Resonance (SCMR) endorsed by the European Association for Cardiovascular Imaging (EACVI). J. Cardiovasc. Magn. Reson..

[13] Ponsiglione A., Gambardella M., Green R., Cantoni V., Nappi C., Ascione R., De Giorgi M., Cuocolo R., Pisani A., Petretta M. (2022). Cardiovascular magnetic resonance native T1 mapping in Anderson-Fabry disease: A systematic review and meta-analysis. J. Cardiovasc. Magn. Reson..

[14] Lukas J., Scalia S., Eichler S., Pockrandt A.M., Dehn N., Cozma C., Giese A.K., Rolfs A. (2016). Functional and Clinical Consequences of Novel α-Galactosidase A Mutations in Fabry Disease. Hum. Mutat..

[15] Reisin R., Perrin A., García-Pavía P. (2017). Time delays in the diagnosis and treatment of Fabry disease. Int. J. Clin. Pract..

[16] Azevedo O., Gago M., Miltenberger-Miltenyi G., Gaspar P., Sousa N., Cunha D. (2017). Mild Left Ventricular Hypertrophy Unravels a Novel Nonsense Mutation of the *GLA* Gene Associated with the Classical Phenotype of Fabry Disease. Cardiology.

[17] Doheny D., Srinivasan R., Pagant S., Chen B., Yasuda M., Desnick R.J. (2018). Fabry Disease: Prevalence of affected males and heterozygotes with pathogenic GLA mutations identified by screening renal, cardiac and stroke clinics, 1995–2017. J. Med. Genet..

[18] Thompson R.B., Chow K., Khan A., Chan A., Shanks M., Paterson I., Oudit G.Y. (2013). T_1_ Mapping With Cardiovascular MRI Is Highly Sensitive for Fabry Disease Independent of Hypertrophy and Sex. Circ. Cardiovasc. Imaging.

[19] Kozor R., Grieve S.M., Tchan M.C., Callaghan F., Hamilton-Craig C., Denaro C., Moon J.C., Figtree G.A. (2016). Cardiac involvement in genotype-positive Fabry disease patients assessed by cardiovascular MR. Heart.

[20] Al-Arnawoot A., O’Brien C., Karur G.R., Nguyen E.T., Wasim S., Iwanochko R.M., Morel C.F.M., Hanneman K.M. (2021). Clinical Significance of Papillary Muscles on Left Ventricular Mass Quantification Using Cardiac Magnetic Resonance Imaging. J. Thorac. Imaging.

[21] Coughlan J.J., Elkholy K., O’Brien J., Kiernan T. (2016). Atypical patterns of cardiac involvement in Fabry disease. BMJ Case Rep..

[22] Nojiri A., Anan I., Morimoto S., Kawai M., Sakuma T., Kobayashi M., Kobayashi H., Ida H., Ohashi T., Eto Y. (2020). Clinical findings of gadolinium-enhanced cardiac magnetic resonance in Fabry patients. J. Cardiol..

[23] Burton J.O., Jefferies H.J., Selby N.M., McIntyre C.W. (2009). Hemodialysis-Induced Cardiac Injury: Determinants and Associated Outcomes. Clin. J. Am. Soc. Nephrol..

[24] Pavlu L., Kocourkova L., Taborsky M., Petrkova J. (2019). Ventricular tachycardia: A presentation of Fabry disease case report. Eur. Heart J. Case Rep..

[25] Nickander J., Cole B., Nordin S., Vijapurapu R., Steeds R.P., Moon J.C., Kellman P., Ugander M., Kozor R. (2023). Increased cardiac involvement in Fabry disease using blood-corrected native T1 mapping. Sci. Rep..

[26] Pica S., Sado D.M., Maestrini V., Fontana M., White S.K., Treibel T., Captur G., Anderson S., Piechnik S.K., Robson M.D. (2014). Reproducibility of native myocardial T1 mapping in the assessment of Fabry disease and its role in early detection of cardiac involvement by cardiovascular magnetic resonance. J. Cardiovasc. Magn. Reson..

[27] Ponsiglione A., De Giorgi M., Ascione R., Nappi C., Sanduzzi L., Pisani A., Dell’aversana S., Cuocolo A., Imbriaco M. (2023). Advanced CMR Techniques in Anderson-Fabry Disease: State of the Art. Diagnostics.

[28] Camporeale A., Moroni F., Lazzeroni D., Garibaldi S., Pieroni M., Pieruzzi F., Lusardi P., Spada M., Mignani R., Burlina A. (2022). Trabecular complexity as an early marker of cardiac involvement in Fabry disease. Eur. Heart J. Cardiovasc. Imaging.

